# Society Meetings

**Published:** 1906-01

**Authors:** 


### SOCIETY MEETINGS.

The Lake Erie Medical Society held its regular quarterly meeting at Dayton N. Y., Friday Janury 26, 1906. Program: I.—Gastroenteritis in children—Report of Case; E. M. Schofield, Jamestown. II—Care and Prevention of Contagious Diseases; G. E. Ellis, Health Officer of Dunkirk. III.—Diagnosis of Cardiac Lesions; Henry C. Buswell, Buffalo. IV.—Discussion and action on the following resolution: Resolved that the best interests of this Society and of the members which compose it, consists in the disbandment of the Lake Erie Medical Society, and the affiliation of its members with the Central New York Medical Association. Officers: president, C. W. Southworth, Forestville; vice-president, B. E. Smith, Angola; secretary and treasurer, E. W. Janes, Hamburg; Program Committee, William A. Putnam, Smith's Mills, and W. M. Ward, North Collins.

The Buffalo Academy of Medicine held meetings during the month of December as follows:

A stated meeting of the Academy, Tuesday, December 12, 1905, at 8:30 P. M. Meeting of the Council at 8:15 sharp. The program of the evening was furnished by the Section of Medicine, as follows: The border line between surgery and intestinal medicine in gastrointestinal diseases, J. A. Lichty, Pittsburg, Pa. The discussion was opened by Dr. A. L. Benedict. A collation was served at the close of the meeting.

*Section of Surgery.*—Tuesday, December 5, 1905, at 8:30 P. M. Program: Bier's treatment by hyperemia. Observations at the surgical clinic in Bonn, Max Breuer. Chronic spasm of the colon, Marshall Clinton.

*Section of Pathology.*—December 19, 1905, at 8:30 P. M. Program: A case of echinococcus cyst of the liver, Edgar R. McGuire; Discussion opened by Dr. Irving P. Lyon. Specimens of leukemia, Charles S. Jewett. Specimen of miliary aneurysm of the brain, with hemorrhage, Herbert U. Williams. Specimen of exopthalmic goitre with thrombosis of the right inominate vein and superior vena cava, Albert E. Woehnert. Specimen of brain tumor, William C. Krauss.

A special meeting of the Academy, Thursday, December 28, 1905, at 8:30 P. M. Program: (a) Moving pictures of Epileptic Convulsions, Walter G. Chase, Boston, Mass (b) Remarks on Epilepsy, William P. Spratling, Craig Colony, Sonyea, N. Y.

---

THE Medical Society of Missouri Valley will hold its next meeting at St. Joseph, Thursday and Friday, March 22 and 23, 1906, under the presidency of Dr. John E. Summers, Jr., of Omaha. The local arrangements are in the hands of Drs. Jacob Geiger, O. B. Campbell and C. R. Woodson, and hospitable St. Joseph extends a hearty welcome to all. The secretary is Dr. Charles Wood Fassett, of Saint Joseph.

---

THE Fifteenth International Medical Congress will be held in Lisbon, April 19 to 26, 1906. The preliminary program and itinerary of the American party which is being organised, describes a most interesting trip at very low cost.

Dr. John H. Musser, of Philadelphia, is chairman of the National Committee, and Dr. Ramon Guiteras, 75 W. 55th street, New York City, is the secretary, to whom all aplications should be addressed.

The sailing date of the American party is April 7, by the North German Lloyd Steamship, Koenig Albert. The arrange- are in the hands of Dr. Chas. Wood Fassett, of St. Joseph, to whom all applications for reservations should be made. In order that proper hotel accomodations may be secured in Lisbon, there should be no delay on the part of those who contemplate attending the Congress.

---

THE fourth International Congress of Examining Physicians for Insurance Companies will be held in Berlin from the 11th to the 15th of September, 1906. Invitations are being sent out for papers. Dr. Alfred Manes, Berlin W., Spichern-Strasse 22, is the general secretary.

